# Probabilistic Modelling of the Food Matrix Effects on Curcuminoid’s In Vitro Oral Bioaccessibility

**DOI:** 10.3390/foods13142234

**Published:** 2024-07-16

**Authors:** Kevin de Castro Cogle, Mirian T. K. Kubo, Franck Merlier, Alexandra Josse, Maria Anastasiadi, Fady R. Mohareb, Claire Rossi

**Affiliations:** 1 Université de Technologie de Compiègne, CNRS, UPJV, GEC, 60203 Compiègne, France; kevin.decastrocogle@cranfield.ac.uk (K.d.C.C.); mirian.kubo@utc.fr (M.T.K.K.); franck.merlier@utc.fr (F.M.); alexandra.josse@utc.fr (A.J.); 2 Bioinformatics Group, Centre for Soil, Agrifood and Biosciences (SABS), Cranfield University, College Rd, Cranfield, Bedford MK43 0AL, UK; m.anastasiadi@cranfield.ac.uk

**Keywords:** curcuminoids, bioaccessibility, food matrix, dietary fibre, screening, regression, Bayesian model, enriched foods

## Abstract

The bioaccessibility of bioactive compounds plays a major role in the nutritional value of foods, but there is a lack of systematic studies assessing the effect of the food matrix on bioaccessibility. Curcuminoids are phytochemicals extracted from *Curcuma longa* that have captured public attention due to claimed health benefits. The aim of this study is to develop a mathematical model to predict curcuminoid’s bioaccessibility in biscuits and custard based on different fibre type formulations. Bioaccessibilities for curcumin-enriched custards and biscuits were obtained through in vitro digestion, and physicochemical food properties were characterised. A strong correlation between macronutrient concentration and bioaccessibility was observed (*p* = 0.89) and chosen as a main explanatory variable in a Bayesian hierarchical linear regression model. Additionally, the patterns of food matrix effects on bioaccessibility were not the same in custards as in biscuits; for example, the hemicellulose content had a moderately strong positive correlation to bioaccessibility in biscuits (*p* = 0.66) which was non-significant in custards (*p* = 0.12). Using a Bayesian hierarchical approach to model these interactions resulted in an optimisation performance of *r*^2^ = 0.97 and a leave-one-out cross-validation score (LOOCV) of *r*^2^ = 0.93. This decision-support system could assist the food industry in optimising the formulation of novel food products and enable consumers to make more informed choices.

## 1. Introduction

As consumers increasingly prioritise health and nutrition, the demand for functional foods and nutraceuticals has surged over the past decade and is projected to reach USD 91 billion by 2031 [1]. These products are specifically formulated to enhance health benefits, incorporating essential nutrients and bioactive compounds such as phytochemicals [2], which are classified as bioactive molecules produced by plants for their protection. Phytochemicals have been categorized into different classes such as carotenoids, phenolic compounds (flavonoids, isoflavonoids, lignin), phenolic acid, phytosterols and phytostanols, tocotrienols, organosulfur compounds, and nondigestible carbohydrates (dietary fibres) [3]. Their consumption has been linked to a potential reduced risk of developing chronic diseases such as cancer and coronary heart disease. They have also been linked to various health benefits, including anti-inflammatory, antioxidant, antibacterial, and antifungal effects [4]. Currently, a regulatory framework is essential to protect consumers from false and misleading claims and to encourage the industry to engage in innovative product development, marketing, and promotion. The future of functional foods depends on the unequivocal demonstration of their efficacy in promoting health [5].

Developing functional foods enriched with phytochemicals poses significant challenges. The first one is related to their susceptibility to degradation from various factors such as heat, pH fluctuations, oxidation, light exposure, and hydrolysis. Careful consideration of processing and storage methods is essential to preserve these molecules in the final product [6,7,8]. Secondly, intrinsic to the food product, molecules that compose the food can interact with each other influencing the digestion, accessibility, release and stability through the digestive process [9]. This can significantly modify the intended biological activity after consumption, potentially invalidating any health benefit claims. Indeed, assimilation rates of the same bioactive compound can vary significantly from one food product to another due to the variability of the chemical interactions that can occur within a food matrix [10]. Food structure may also have an influence on the release of the bioactive compounds [11]. For these reasons, there is growing interest in understanding and mastering the impact of food matrices on the bioaccessibility of phytochemicals. Many functional foods, in addition to incorporating phytochemicals, are also fortified with health-promoting nutrients such as proteins or fibres [12]. Studies suggest that these nutrients may reduce the absorption of phytochemicals, potentially affecting their health benefits. For instance, proteins, fibres, and minerals have been found to diminish the bioavailability of flavonoids in both in vitro and in vivo studies [13]. However, other studies present conflicting findings, and there is no consistent pattern regarding the impact of proteins or fibres on the availability of phytochemicals [14]. This variability appears to be influenced by the specific characteristics of the proteins, fibres, and phytochemicals involved. Additionally, the complexity of chemical interactions occurring within food products makes it challenging to predict outcomes accurately. This highlights the need for innovative approaches to systematically investigate how food matrices impact phytochemical release and to select the most suitable type of nutrients for formulations while ensuring that targeted health claims are maintained.

Turmeric, a spice derived from the dried rhizome of the *Curcuma longa* plant, contains numerous phytochemicals. Notably, curcuminoids play a central role and can constitute up to 10% of dry turmeric powder. While over 50 curcuminoids have been identified in turmeric, the major ones found in commercial products are curcumin (62–90%), demethoxycurcumin (9–23%), and bisdemethoxycurcumin (0.3–14%) [15]. Curcumin has been extensively studied [16] for its biological activities in a large number of cell culture-based and animal studies. They reported strong anti-inflammatory and anti-oxidant and neuroprotective properties [17]. Curcumin exhibits various preventive properties against a range of disorders, including rheumatoid arthritis, diabetes mellitus, cardiovascular issues, and cancer [18]. Nevertheless, the potential of incorporating dietary curcumin as a feed additive has been highlighted. An example is seen in a study where supplementing quails’ diets with curcumin (at a concentration of 200 mg/kg) resulted in enhanced egg quality. This improvement was attributed to the promotion of lipid metabolism and the augmentation of antioxidant activity in the quails [19]. Despite its numerous health benefits, the preventive or therapeutic use of curcumin in humans for treating various diseases is constrained due to challenges such as its low solubility in water, rapid metabolism, rapid elimination, and, consequently, limited bioavailability [20]. Many strategies towards improving the bioavailability have been developed in recent years, most of which focus on transport systems around the use of nanoparticles, optimised curcumin formulations (using fibres or oil emulsions), complexing/encapsulating, or even through the use of adjuvants such as piperine, strategies that have achieved bioavailability increased between 2- and 100-fold [20]. Most of these strategies focus on increasing the aqueous solubility of curcumin so that it is more accessible by the time it reaches the sites of intestinal uptake. Bioaccessibility, which refers to the fraction of a compound that is released from its carrier throughout the gastrointestinal journey in a form which is absorbable by the intestine, has been discussed to be the main limiting factor in absorption of fat-soluble compounds [21]. Therefore, it is usually the aspect of absorption that receives the most attention for increasing their overall bioavailability. Bioaccessibility can be efficiently screened for numerous conditions using in vitro methods such as INFOGEST’s consensus model [22]. Through this in vitro approach, the presence of emulsified lipids has been associated with heightened bioaccessibility of carotenoids, demonstrating a corresponding increase in their bioavailability under similar conditions [23].

Incorporating water-insoluble phytochemicals into food and improving their release is challenging. Curcumin and other curcuminoids from turmeric are excellent candidates for demonstrating how machine learning can model the impact of food formulation on the bioaccessibility of these compounds and predict the best formulations. It has been shown that curcuminoids have an affinity for certain types of fibres, such as sugar beet pectin, a soluble prebiotic fibre which can improve the shelf life of curcumin within the food matrix [24]. Animal and human studies have shown that curcuminoids’ impregnation into a soluble fibre isolated from the fenugreek spice or the formation of complexes with cellulose derivates enhances its bioavailability [25]. Moreover, there is currently great public interest in consuming dietary fibre due to several health benefits they are associated with [26,27]. Fibre supplements enable the production of a wide range of fibre-fortified foods, which can be used as a source of variability to study the impact of food formulation on curcuminoid release.

Statistical inference methods are a powerful tool for the goal of unravelling the factors governing phytochemical absorption. Machine learning is gaining popularity in the food industry [28], and its methods have shown promise for the prediction of drug oral bioavailability and post-consumption food effects [29]. The aim of this work is to demonstrate the efficacy of modelling the bioaccessibility of phytochemicals based on food formulation. Here, we examined how different types and sources of fibres could affect the bioaccessibility of curcuminoids from *Curcuma longa* extract in two distinct food matrices. For this purpose, several dietary fibre sources were supplemented to custards and biscuits as the source of variation between the formulations, and we assessed the impact of multiple matrix properties on curcuminoid bioaccessibility. The experimental data were used to develop a decision-support system consisting of a predictive mathematical model that could infer the most optimised formulation process for enhancing curcuminoid bioaccessibility in custards and biscuits. 

## 2. Materials and Methods

### 2.1. Preparation of Food Matrices

The custards and biscuits used in this study were prepared as described in Table 1. Egg yolks were prepared from egg yolk powder (Gallia, Louis François, France) and rehydrated according to the manufacturer’s instructions. The egg yolk percentages mentioned below are always after reconstitution. Custards were made with corn starch instead of wheat flour to ensure a custard-like consistency. The base recipes for the custards and biscuits contained equal amounts (mass) of solid ingredients. 

For custard, all ingredients were mixed while taking care to pour the milk last into a HotmixPro thermal mixer (Vitaeco, Modena, Italy). Custards were cooked for 10 min at an agitation of 200 rpm at 85 °C and then stored after cooling at 4 °C or −18 °C until use. The biscuits’ dough was mixed using a Kenwood Mix KMX50 (Yokohama, Japan) until homogenization. Thirty grams of dough was shaped into a biscuit form in a circular mould 10 cm in diameter and 2 cm in height. Six biscuits were prepared in this manner for each formulation. The shaped dough was baked for 30 min at 160 °C in a professional oven (Venix G07M, Veneto, Italy). Biscuits were stored in the dark in sealed bags at room temperature for short period of time until further analyses.

The compositions of the fibre-fortified custard and biscuits are also given in Table 2. The list of fibres used and their nutritional composition, according to the products’ specifications provided by suppliers, are also presented in Table 2. The types of fibres of each supplement were taken from the literature [30]. Fibre addition was limited to 6% to avoid potentially negative sensory properties (pungent flavours or too hard/soft texture) for potential consumers, as was observed in a small test cohort in the lab. The following soluble fibres were used: a soluble inulin powder Orafti^®^ (Beneo GmbH, Mannheim, Germany) from chicory; a soluble fibre Nutriose^®^ FM06 (Roquette) from maize; a soluble citrus peel fibre (Ceamfibre 7000, CEAMSA, Pontevedra, Spain); two different carrot fibres (FST 00007 KaroPRO 1-26 and FST 00018 KaroPRO SG, FoodSolutionsTeams GmbH, Hettingen, Switzerland) containing the same amount of both soluble and insoluble fibres but displaying different physical properties; an insoluble apple fibre (FST 00224 ApplePRO 60+, FoodSolutionsTeams GmbH); an insoluble yellow pea fibre (Pea fibre 50 M Roquette frères, Lestrem, France); an insoluble wheat fibre (Vitacel WF200, JRS, Rosenberg, Germany); and an insoluble microcrystalline cellulose (Redwells^®^, London, UK) from wood pulp. 

Custards were enriched with 35.6 mg/100 g of curcuminoid powder (curcumin content approx. 75%, C 1386, CAS 458-37-7, Sigma-Aldrich, Saint Quentin Fallavier, France), whilst biscuits were enriched with 106.7 mg/100 g of curcumin powder prior to cooking in both cases. Liquid matrices (water and milk) were prepared by dissolving 35.6 mg/100 g of curcuminoid powder in room temperature water and milk by vortexing. Food preparation protocols were carefully observed (i.e., oven tray height) in order to ensure reproducible conditions. No significant degradation in curcumin was observed during or after processing. 

### 2.2. Static In Vitro Digestion

Static in vitro digestion was carried out following the guidelines established by the updated INFOGEST consensus protocol [22], with slight modifications. Bacterial α-amylase from Megazyme (Bray, Ireland) was used as a replacement for human salivary amylase at the same activity, and rabbit gastric lipase was not employed in the gastric phase. Every food formulation was digested a minimum of 6 times. For simulating digestion of custard and liquid matrices, 5 g of food was digested, as recommended. In the case of biscuits, this required mixing 1.667 g of previously ground biscuits (Blender Fruit Sensation, Moulinex, Paris, France) with 3.333 g of ultrapure water to obtain a tomato paste-like consistency, as recommended by the methodology and to ensure both custards and biscuits had similar concentrations of solid matter (~1.15 g) and curcuminoids (curcumin powder from Sigma Aldrich) (~1.78 mg) in the digested samples. 

### 2.3. Curcuminoid Bioaccessibility Quantification

Curcuminoid bioaccessibility was quantified following the method applied previously to buttermilk [31] with slight modifications. Ethanol and chloroform (>98% pure) were purchased from VWR Chemicals (Radnor, PA, USA). Ultrapure water (from Milli-Q^®^ system) was used throughout the experiment. 

A total of over 6 biological replicates for each food formulation was generated in this manner. Bioaccessibility was calculated as:

(1) $$bioaccessibility\;\left(\%\right)=\frac{\left(curcuminoids\;in\;bioaccessible\;fraction\right)}{\;\left(curcuminoids\;in\;total\;fraction\right)}\times 100\%$$

### 2.4. Curcuminoid Quantification by HPLC-DAD 

Quantification of curcumin (CUR), demethoxycurcumin (DMC), and bis-demethoxycurcumin (BDMC) was performed by liquid chromatography on an HPLC Agilent 1290 equipped with a DAD detector. The HLPC analyses were performed using a Thermo Hypersil Gold C18 column (100 × 2.1 mm, 1.9 µm, 175 A) at 35 °C. Eluents A and B consisted of 0.1% (*v*/*v*) formic acid in deionized water using a Milli-Q system (Millipore, Molsheim, France) and 100% acetonitrile (with UPLC-MS grade from Biosolve Chimie, Dieuze, France), respectively. The gradient program started with 2% B, then was increased to 98% B over 18 min and held at 98% for an additional 2 min before returning to the initial conditions and remaining constant for 3 min. The flow rate was 0.400 mL/min and the injection volume was 5 µL. Detection was monitored at 220, 262, 280, and 430 nm and in the range of 210 to 640 nm with 2 nm steep. 

Quantification of curcumin (CUR), demethoxycurcumin (DMC), and bis-demethoxycurcumin (BDMC) was performed by liquid chromatography–high-resolution mass spectrometry (LC-HRMS) on an HPLC Agilent 1290 with DAD connected to hybrid quadrupole time of flight (Q-TOF) Q-TOF 6538 (Agilent Technologies, France). HPLC analyses were performed using a Thermo Hypersil Gold C18 column (100 × 2.1 mm, 1.9 µm, 175 A) at 35 °C. Eluents A and B consisted of 0.1% (*v*/*v*) formic acid in deionized water using a Milli-Q system (Millipore, Molsheim, France) and 100% acetonitrile (with UPLC-MS grade from Biosolve Chimie, Dieuze, France), respectively. The gradient program started with 2% B, then was increased to 98% B over 18 min and held at 98% for an additional 2 min before returning to the initial conditions and remaining constant for 3 min. The flow rate was 0.400 mL/min and the injection volume was 5 µL. Detection was monitored at 220, 262, 280, and 430 nm and in the range of 210 to 640 nm with 2 nm steep. All compound responses were measured using negative electrospray ionisation (ESI) and calibrated externally. The ESI Gas Temp was 350 °C, Vcap—3800 V; the drying gas was set at 10 L/min, and the Nebuliser was set at 30 psig. The fragmentor was set at 140 V. The HRMS spectrum was registered at 2 Hz in the mass range of 100 to 3000 *m*/*z*. MassHunter software (B.07) and MSDIAL 4.9 [32] were used for data processing. 

### 2.5. Characterization of Curcuminoid-Enriched Formulations 

#### 2.5.1. Food Digestibility Assays

The degree of starch hydrolysis of the food samples after digestion was characterised using the Total Starch HK Assay Kit (K-TSHK) from Megazyme (Bray, Ireland). The degree of proteolysis was assessed using the o-phthalaldehyde (OPA) method, which was used for assessing milk protein proteolysis [33], but using glycine instead for building the standard curve.

#### 2.5.2. Oil- and Water-Holding Capacities of Fibres

The oil-holding capacities of the different fibres were characterized by adding 1 g of each fibre in a 50 mL centrifuge tube, then adding 8 mL of a mix of colza and sunflower oil and vortexing. The tubes were left to stand for 30 min at room temperature. The samples were centrifuged for 15 min at 1057× *g*, supernatants were discarded, and the excess of oil on the wall of the centrifuge tube was absorbed using absorption papers. The tubes were weighed and compared to the initial weight prior to adding oil. Each assay was performed in triplicate. The OHC was calculated using the following equation:

(2) $$OHC\left(g/g\right)=\frac{m_{2}-m_{1}}{m_{0}}$$

where $m_{1}$ refers to the centrifuge tube; $m_{2}$ refers to the mass of the centrifuge tube plus the oil holding sample; and $m_{0}$ refers to the dry fibre mass.

Similarly, the water-holding capacities of insoluble fibres were measured (in triplicate) by mixing 1 g of each fibre with 30 mL of ultrapure water in a centrifuge tube, vortexing, and letting it stand for 16 h. Unretained water was removed from each sample by filtration on polypropylene membranes (0.2 µm pore size, GH Polypro, Pall, New York, NY, USA) using a Büchner filtration system (Millipore, Burlington, MA, USA). The water-holding capacity was calculated as follows:

(3) $$OWC\left(g/g\right)=\frac{m_{2}-m_{1}}{m_{0}}$$

where $m_{1}$ refers to the mass of the wet membrane, $m_{2}$ refers to the mass of the wet membrane plus the oil-holding sample, and $m_{0}$ refers to the dry fibre mass. 

#### 2.5.3. Bulk and Tapped Density of Fibres

Bulk density was determined by filling a 50 mL graduated cylinder with each fibre source and weighing. The weight of the empty cylinder was subtracted from the total weight. Bulk density was calculated as:

(4) $$bulk\;density\;\left(g/\mathrm{mL}\right)=\frac{fibre\;weight\;\left(g\right)}{\;50\left(\mathrm{mL}\right)}$$

Tapped density was measured by weighing 10 g of each fibre in a graduated 50 mL cylinder and repeatedly tapping (dropping from a height of 10 cm) the cylinder until the fibre source did not compact any further. The volume was recorded. Tapped density was calculated as:

(5) $$tapped\;density\;\left(g/\mathrm{mL}\right)=\frac{10\;\left(g\right)}{\;fibre\;volume\;\left(\mathrm{mL}\right)}$$

#### 2.5.4. Biscuit-Breaking Force 

Biscuits had previously been cut into a 3.5 by 10 cm squircle. The hardness was measured as the force required to break a biscuit in half by the centre using the three-point break method using a CT3 Texture Analyzer (Brookfield Engineering, Middleboro, MA, USA) with a TA-7 (knife-edge) probe. The analysis was set at one compression cycle and a speed of 0.5 mm/s. The maximum breaking force in g was recorded and used as a descriptor of biscuit hardness. The trigger force load was 5 g. Measurements were made in triplicate.

#### 2.5.5. Textural Analysis of Custard

The firmness and stickiness of the custards was determined using the CT3 Texture Analyzer with a TA-10 probe. The custards were submitted to one cycle of 20 mm distance compression at a speed of 0.5 mm/s. The firmness was determined as the force necessary to attain the 20 mm distance within the food product. The adhesion was defined as the work necessary to overcome attractive forces between surface of the food and materials as the probe retracted. The stickiness was measured as the maximum negative load of the probe after going back up from a 20 mm depth in the food sample.

The viscosity was measured in triplicate with a Lamy RM200 viscometer (Lamy Rheology, Champagne-au-Mont-d’Or, France) equipped with a MK-DIN 12 measuring body at 20 °C. Once the mobile MK-DIN-2 had entirely plunged into the cream, the measurement was performed with a speed gradient increase from 2 to 100 s^−1^ for 60 s. The gradient of 100 s^−1^ was maintained for 10 s before inverse deceleration. The data were obtained from the rheometer’s computer software, and viscosities evaluated at 100 s^−1^ were reported.

#### 2.5.6. Fibre Colour Determination

Fibre colour was assessed with a colorimeter (PCE-CSM 4, PCE Instruments, Meschede, Germany) in a CIE L*a*b scale. The L* value (lightness index scale) ranged from 0 (black) to 100 (white); a* indicates the red (+a*) or green (−a*) value and the b* refers to the yellow (+b*) or blue (−b*) value. Measurements were made in triplicate. 

#### 2.5.7. Curcuminoids Relative Binding Capacity of Fibre Sources

A quantification assay was devised to gauge the level of curcuminoid entrapment by the different fibres. This assay relies on determining the minimum concentration of curcuminoids in an aqueous solution in contact with fibres that is necessary to start to detect unbound curcuminoids, thereby elucidating the efficacy of curcuminoid entrapment. This assay cannot be performed with soluble fibres. 

Briefly, 1 g of each fibre was added to a 50 mL centrifugation tube with 20 mL of ultrapure water. Concentrated stock solutions of curcuminoids in DMSO (1–10 mg/mL) was added in small and incremental volumes (20–500 μL). The tubes were mixed by vortexing and subjected to shaking incubation (Infors HT Multitron, Infors AG, Bottmingen, Switzerland) lasting 2 h at 200 rpm and 37 °C in the dark. Afterwards, an aliquot of 2 mL was extracted and centrifuged for 10 min at 17,000× *g*. The supernatant was measured at 435 nm in a spectrophotometer (Analytik Jena Specord 205, Jena, Germany) against a blank of the same fibre source and treatment, but with no added curcumin, and the concentration of curcuminoids was quantified using a calibration curve ranging from 5–40 mM curcuminoids in DMSO/water. This same procedure was repeated while adding different concentrations of curcuminoids in order to build a curve correlating the total concentration of curcuminoids added to the sample to its respective unbound concentration. The maximum concentration of unbound curcuminoids under a limit of detection (LOD) threshold of 2.5 mM was presented as the relative binding capacity. These LOD determinations were performed in separate experiments; a minimum of 5 different concentrations per fibre per experiment were tested (one replicate per concentration, Figure S1).

#### 2.5.8. Particle Size Distribution Measurement

The particle size distribution (PSD) of the fibre sample was determined using laser light scattering with a Mastersizer 2000 device, with a Scirocco 2000 accessory, from Malvern Instruments (Malvern, UK). Each measurement was carried out in triplicate. The Brouckere diameter (*D*_4.3_), also called the volume-weighted mean diameter, was determined according to Equation (6):

(6) $$D_{4.3}=\frac{\sum n_{i\;}D_{i}^{4}}{\sum n_{i\;}D_{i}^{3}}\;$$

where *D_i_* is the average particle diameter and *n_i_* is the number of particles. 

### 2.6. Curcuminoid Bioaccessibility Model Development

#### 2.6.1. Data Description

A total of 9 fibre supplements and one non-supplemented formulation were tested for two matrix types (biscuits and custards) using 2 technical replicates for each of the 132 replicates of digestions of curcuminoid-enriched foods. These were used as samples in the training and validation of the bioaccessibility model. A total of 29 candidate predictors of bioaccessibility were collected and are enumerated in Supplementary Table S1. Missing values were removed in the analyses or inputted using scikit-learn’s IterativeImputer and its default parameters [34]. Outliers were identified using the interquartile range approach (IQR) and Grubbs’ test for outliers and removed accordingly prior to exploratory data analysis and model development. The final dataset comprised 130 biological replicates; only 2 biological replicates were removed.

#### 2.6.2. Feature Selection

The fibre supplement’s colour properties were removed from the dataset prior to feature selection after dismissing the possibility that they were interfering with the target variable (bioaccessibility), as they were poor predictors of bioaccessibility on their own and did not substantially impact the performance of the final model when added as covariates. 

Afterwards, several methods were employed to select the most appropriate independent variables explaining the impact of formulation on bioaccessibility. First, Spearman’s correlation coefficient was computed for the bivariate comparison between all independent variables and bioaccessibility, without scaling. The presence of a statistically significant correlation between feature pairs was assessed using Spearman’s correlation coefficient (ρ) test over Pearson’s, as the normality of some variables could not be asserted due to a lack of sufficient data, as well as for the purpose of considering the possibility of non-linear correlations. 

Together with the bivariate correlations, wrapper/embedded methods were employed. On one hand, the autofeat [35] Python library was used to select the most meaningful features for each matrix type. The Boruta algorithm [36] was also used to select features in a separate experiment. Once the final framework for the model had been decided (details in Section 2.6.4), it was fitted to all the features that had been highlighted by Boruta or autofeat; Laplacian priors centred at 0 were placed on each of the regression coefficients to perform feature selection in a manner equivalent to LASSO selection [37]. 

The most significant features according to the posteriors of the Laplacian prior model were used as a basis for the final model. 

#### 2.6.3. Model Training

A linear regression Bayesian hierarchical model was fitted to the data, with partially pooled covariates. Due to the lack of previous domain knowledge regarding a choice of suitable priors for the model, a data-driven choice was made for the selection of features (Section 2.6.1) and their priors. Macronutrient content was used as a pooled covariate (meaning all the j sample groups would be modelled with a single β model regression parameter) as it presented a very strong correlation to bioaccessibility across all formulations (ρ=0.89), and ordinary least squares regression (bioaccessibility=Slope×Macronutrient content+Intercept) produced the μ location parameter values for the normally distributed priors of its pooled intercept and coefficient. A scale parameter σ=10 was assigned to facilitate the inference procedure from exploring other values around this mean centre. 

With this constraint, the non-pooled covariates (each j group had a different parameter $\beta_{kj}$ for each k variable) were selected by trial-and-error as described in Section 2.6.1. All tested feature combinations were assigned a normal prior distribution for each $\beta_{kj}$, with the hyperparameters being determined automatically by the Bambi package [38]. In mathematical notation, the different models trialled shared the following specification, parting from the linear regression equation:

(7) $$\mu_{ij}=\alpha +\beta_{macronutrient\_content}X_{i}+\sum_{k=1}^{K}\beta_{kj}X_{ij}+\varepsilon$$

where $\mu_{ij}$ represents the centre of the predicted distribution of the outcome variable for sample i in group j (e.g., the peak of the predicted bioaccessibility of pea fibre biscuit); α represents the intercept of linear regression; $X_{i}$ and $X_{ij}$ represent the values of the pooled and non-pooled covariates, respectively; and ε represents random error.

The response variable Y was assumed to follow a Student’s t distribution (likelihood), with the following specifications:

(8) $$Y_{ij}\;\sim \;\mathrm{Student}\left(\nu_{j},\mu_{ij},\;\sigma_{j}\right)\;\sigma_{j}\;\sim \;\mathrm{HalfNormal}\left(\sigma =1\right)\;\nu_{j}\;\sim \;\mathrm{Gamma}\left(\alpha =2,\;\beta =0.1\right)$$

where $\nu_{j}$ represents the degrees of freedom and $\sigma_{j}$ the scale parameter of each group. The pooled intercept and “*macronutrient_content*” coefficient priors were predefined with a normal distribution:

(9) $$\alpha \;\sim \;N\left(\mu =13,\sigma =10\right)\;\beta_{macronutrient\_content}\;\sim \;N\left(\mu =40,\sigma =10\right)$$

And during the trial-and-error process of using different non-pooled covariates, all group-specific priors were equally assigned:

(10) $$\beta_{kj}\;\sim \;N\left(\mu_{\beta_{k}}=0,\sigma_{\beta_{k}}=\mathrm{HalfNormal}\left(\sigma =\tau_{\beta_{k}}\right)\right)$$

where each $\tau_{\beta_{k}}$ were automatically determined by Bambi. The PyMC framework [39], through Bambi’s API, was employed to carry out parameter estimation and inference using the NUTS method (+jitter and adapt) with 2000 tuning iterations and 2000 sample draws across 4 chains. No divergences were reported at inference time at a target acceptance rate of 0.95. The ArviZ [40] library facilitated pre- and post hoc analysis of the fitted model during the trial-and-error process of evaluating the candidate models.

#### 2.6.4. Model Validation and Assessment

The procedure of estimating the generalization capacity of the best-fitting models followed the Leave-One-Out Cross-Validation (LOOCV) strategy. In this procedure, the observations of the same formulation were held out as a validation sample and the models were trained on the remaining observations. To test the robustness of the models, all possible combinations were tested; all the formulations were left out individually for a given iteration. This was performed with the purpose of visualising the comparison between predicted and observed bioaccessibilities, as well as to compute a practical measure of loss for each model.

In each iteration, the test formulation’s average bioaccessibility was compared to the formulation’s posterior predictive distribution average- and minimum-width Bayesian credible interval (BCI) (also called highest-density interval, HDI). The mean absolute error (MAE), root mean squared error (RMSE), and coefficient of determination (R-squared, R2, and $r^{2}$ used equivalently) were used as performance evaluation metrics in this validation case as well.

## 3. Results and Discussion

### 3.1. Food Digestibility Assessment 

No traces of starch were detected in the digested food samples, so it was inferred the digestions had been successfully completed in that regard, and this was considered as an indicator of a representative simulated digestion. OPA assays showed the released glycine equivalents increased 8-fold after digestion (from 0.8 mmol glycine equivalents per mg of protein to around 6.5), which constituted a similar proteolysis degree to what was expected when compared to the results reported by the protocol authors [33]. However, there was no significant difference in glycine equivalents’ release among the subset of formulations tested; hence, the degree of proteolysis was not included as a variable of study. These findings indicated that the degree of macronutrient digestibility would not provide significant predictive power towards bioaccessibility, so the lipolysis degree was not assessed. Fat content was highly homogenous throughout the different formulations (i.e., all custards had similar amounts of fat content, and the same for biscuits); therefore, fat digestibility was dismissed as a predictor in the case of this experiment design. Future experiments can address this in more detail, as the presence and type of fat in meals is known to exert a significant effect (although not linearly correlated) on the release and absorption of fat-soluble compounds in the food matrix [41].

### 3.2. Validation of the Spectrophotometric Method to Quantify Total Curcuminoids Content

To simplify the evaluation of curcuminoid content, these compounds were quantified using UV spectrophotometry at 435 nm. To validate this approach, samples of biscuits and custards, both with and without curcumin supplementation, were analysed using HPLC-DAD, with curcuminoid identification by mass spectrometry and electrospray ionization. The UV chromatogram at 430 nm (Figure S1) primarily reflected the presence of the three main curcuminoids: BDMC, DMC, and CUR, consistent with the manufacturer’s specifications (Table S1). Peak attribution in the 430 nm UV chromatogram was confirmed via high-resolution tandem mass spectrometry and comparison with MS/MS databases.

To further validate the quantification of total curcuminoids using spectrophotometry, curcuminoid levels in custard and biscuit samples without fibre supplementation were measured using both spectrophotometry and HPLC-UV. Due to the small sample sizes and the bimodal distribution of bioaccessibilities in these samples, the Mann–Whitney U test was employed to determine if the bioaccessibility measurements from HPLC-DAD (*n* = 8) and spectrophotometry (*n* = 14) belonged to the same population. The test statistic (U) was −52.0 with a *p*-value of 0.82. With the significance level set at 0.05, these results indicate no significant difference between the two groups, thus validating the spectrophotometric quantification of curcuminoid’s bioaccessibility against the HPLC-DAD method. Moreover, HPLC-DAD indicated that the ratios of major curcuminoids in the turmeric powder were 70–73% curcumin, 20–21% demethoxycurcumin, and 7–9% bis-demethoxycurcumin. These values are in line with the literature [15]. After digestion, these ratios were similar: 75–77% curcumin, 18% demethoxycurcumin, and 5–7% bis-demethoxycurcumin.

### 3.3. Curcuminoids Bioaccessibility in Food Formulations

**Figure 1:**
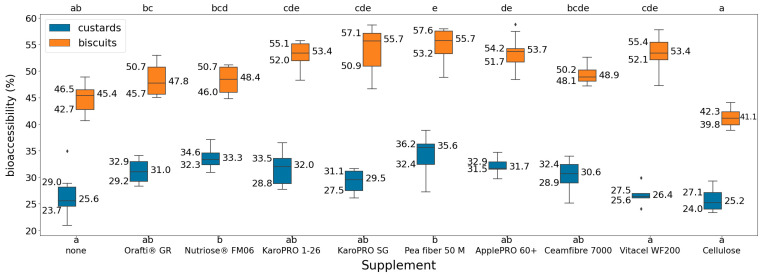
Boxplots of the bioaccessibilities measured for each formulation. The fibres are ranked from the lowest to the highest insoluble fibre content (left to right). Each formulation consisted of a minimum of 5 biological replicates after outlier removal. The compact letter display was computed separately for custards (bottom) and biscuits (top). Formulations with different letters presented statistically significant differences to one another (according to Tukey tests).

First, the bioaccessibility of curcuminoids was measured in water and milk. The obtained values resembled those obtained by the protocol authors [31] under similar conditions, in the 11.4–26.7% range compared to our 12.4–25.9% range. The curcuminoid bioaccessibility for the custard and biscuit formulations are displayed in Figure 1; they were in the range of 20.9–38.8% for custards and 38.8–58.8% for biscuits. To the best of our knowledge, there is no comprehensive study available in the literature regarding the bioaccessibility of curcumin in these types of complex food matrices. However, it has been reported that curcuminoid’s bioaccessibility can vary from as low as 0.4–0.8% up to 25%, 50%, and as high as 91% with optimization such as nanoemulsions, nanoparticles, or cyclodextrines [42]. As a general trend, our results show that the presence of fibre increases bioaccessibility, except in the case of cellulose. Thus, dietary fibre enrichment has the potential to enhance the curcumin release from the studied food formulations. However, at first glance, no clear trend can be identified based on the nature of the fibre (soluble or insoluble). 

To evaluate the assumption of normality of the observed bioaccessibility distributions, the Shapiro–Wilk test was conducted with a significance level of α=0.05. The assumption of normality was not rejected for custard bioaccessibilities (W=0.99, p=0.70) nor biscuit bioaccessibilities (W=0.98, p=0.26) when considered independently, but it was rejected when considering them altogether ($W=0.93,\;p=4.27\cdot 10^{-16}$). Individually, every formulation was also assumed to have normally distributed bioaccessibilities, and visual inspection of QQ plots and Shapiro–Wilk tests did not reject the assumption. However, the distribution of the bioaccessibilities across all formulations was bimodal, with biscuits having higher bioaccessibilities than custards. In any case, homoscedasticity was assumed across all formulations’ variances, and Levene’s test (less sensitive to departures from normality compared to other tests) did not reject this hypothesis ($F\left(19,110\right)=0.99,\;p=0.47$).

A two-way analysis of variance (ANOVA) was performed to assess the effects of matrix type and its interaction with fibre supplements on bioaccessibility. The normality of the residuals was visually inspected and confirmed with Shapiro–Wilk (W=0.99, p=0.61) as well. There was a statistically significant interaction between the effects of matrix type (custard or biscuit) and fibre supplement ($F\left(1,9\right)=403.56,\;p=1.81\cdot 10^{-5}$). This interaction had a partial eta-squared of $\eta^{2}p=0.28$, indicating a small effect size on bioaccessibility; within each matrix type, the different supplements had statistically significant effects on bioaccessibility. Simple main effects analysis also showed that matrix type ($p=1.86\cdot 10^{-63}$) had a statistically significant impact on bioaccessibility. Matrix type had a larger effect size ($\eta^{2}p=0.92$) than the interaction. Within each matrix type, the specific formulations that presented significant differences were investigated thereafter with Tukey’s HSD test (confidence level of 95%). The pairwise formulations that presented statistically significant differences to each other were not the same in both matrix types, suggesting that the pattern of impact effects diverged according to this grouping. These are included as a compact letter display in Figure 1.

These preliminary findings offer encouraging results towards the possibility of systematically modelling the effect of food matrix effects on nutrient bioaccessibilities through screening studies. Just by considering the type of food matrix and the usage of supplements, these significant differences, if explained by the known properties of the food, could offer valuable insights that could aid in optimising the design of functional foods.

Biscuit formation led to a significant increase in the curcuminoid bioaccessibility compared to custards. An opposite trend was observed for lutein when comparing its release from custard and biscuits, i.e., a higher released from custard than from biscuits [11]. Recently, Zhang et al. reviewed the effects of food matrix structure and components on carotenoid bioaccessibility [43]. The bioaccessibility and release of bioactive molecules are strongly influenced by molecular interactions within the food matrix. For lipophilic compounds, interactions with lipid phases or droplets are a major factor driving their transfer from the digesta. Solubilisation has been theorized in the past as the most important factor impacting curcuminoid bioaccessibility and posterior absorption [44]. However, in complex foods, predicting the hydrolysis profile of macronutrients during digestion remains challenging, and no systematic correlation between bioactive compounds and food matrix types has been established to date.

To better characterise the factors influencing bioaccessibility across formulations, a more extensive investigation of the specific properties of the foods was carried out.

### 3.4. Curcuminoid Bioaccessibility—Explaining Matrix Effects

We investigated whether some physical properties of fibre or food could be correlated with the bioaccessibility changes. Regarding the properties of the fibres, their oil-/water-holding capacity, bulk/tapped density, curcumin-binding capacity in water, particle sizes, specific surface areas, and colour were measured. Likewise, food texture elements such as biscuit hardness and custard viscosity, firmness, and stickiness were assayed. Data are provided in Table 3 and Table 4. These empirical measurements were complemented with theoretical nutritional composition data, such as the contents of protein, carbohydrates, fat, water, ash, and types of fibre (cellulose, hemicellulose, pectin, lignin, fructans, and dextrin—Table 2 and Supplementary Table S3). Table 5 contains the Spearman’s rank correlation coefficients between the candidate explanatory features and bioaccessibility. Factors were classified as food matrix properties or fibre properties. Only total macronutrient content (sum of protein, carbohydrates, and fat ratios) was considered for comparing the two different food matrices, as fibre contents were the same in all formulations. As the pattern of impact effects on bioaccessibility depended on the matrix type, fibre properties had to be inspected independently for each group. None of the correlations of these variables were found to be statistically significant (*p*-value = 0.05) when considering all samples together. 

The capacity of the fibre to trap curcumin was initially believed to have a strong negative correlation to bioaccessibility, as the fibres tested for this property presented mostly insoluble fibre, which would not be digested and would precipitate, therefore negatively impacting bioaccessibility. However, there was only a weak negative correlation observed in custards, and the opposite was observed in biscuits. Moreover, these correlations were not statistically significant (*p*-value = 0.05). It was also of note that none of the texture properties correlated with bioaccessibility. 

On the other hand, as was evidenced by ANOVA tests, the matrix type had a significant impact on bioaccessibility, and this was captured by very strong correlations (ρ=0.89) between bioaccessibility and macronutrient content. Due to not detecting a single property strongly correlated to bioaccessibility within each matrix type (custards and biscuits), an approach to modelling that could consider the complex intra- and extra-group variance was followed. 

### 3.5. Modelling Bioaccessibility as a Function of Food Formulation Properties

The elucidation of factors influencing bioaccessibility in real foods enriched with fibre supplements, a type of food matrix more complex than liquids or pure carriers, required testing several statistical techniques and data modelling workflows to determine which was optimal for the task. 

Initially, principal component analysis (PCA) of all candidate predictors of curcuminoid bioaccessibility was performed (Supplementary Figure S3), followed by a PCA for the predictor subset employed in the 6-parameter model as per the feature selection process explained in Section 2.6.2. (Supplementary Figure S4). Regarding the predictive model development, after considering numerous frequentist algorithms such as ensembles of decision trees, regularized regression, support vector machines, or k-nearest neighbours, Bayesian hierarchical linear regression was considered the most suited to the modelling task, and models built with several feature combinations were evaluated based on prediction performance. The models were well fitted to the data and provided valuable information regarding what factors could be affecting bioaccessibility the most. The results are compiled in Table 6. 

In total, 20 formulations of custards and biscuits comprising 130 bioaccessibility replicates were used to train the probabilistic model. Liquid matrices (water and milk) were included as a benchmark to demonstrate how the use of macronutrient content as a predictor could produce reasonably accurate predictions for formulations beyond custards or biscuits, even with imbalanced datasets (performance of liquid matrix group is not reported as only two samples were available). By using only this predictor and an intercept, all formulations were well fitted ($r^{2}$ of 0.94); however, there was underfitting in each matrix type, with an average $r^{2}$ of only 0.42. When excluding liquid matrices from model fitting, error metrics were reduced by 14% on average, as liquid matrices could still influence the inference of pooled parameters. 

In order to improve the performance of the model within each matrix type, additional input variables to train the algorithm were considered. With the addition of soluble fibre as a non-pooled covariate (4-parameter model), the goodness of fit was improved (average $r^{2}$ of 0.55 for both matrix types); however, it was with the additional inclusion of hemicellulose (6-parameter model) that it was considered optimized (average $r^{2}$ of 0.72). The addition of a third non-pooled feature (texture properties, 8-parameter model) only increased the goodness-of-fit $r^{2}$ to 0.77, and other non-reported combinations did not perform significantly better. 

The improvement in the goodness of fit by including extra predictors was substantial. In cases such as this, the chance of producing an overfitting model grows with each predictor. The overfitting phenomenon can be evaluated with cross-validation experiments such as the Leave-One-Out Cross-Validation (LOOCV) experiment, in which the performance of the model with unseen samples is estimated by training the model iteratively with all samples except one, and for this left-out sample, the difference in predicted bioaccessibility versus observed bioaccessibility is collected as an error. This is repeated for all samples, one by one, and error metrics are summarised and used as model performance metrics. 

In this case, the improvement in the goodness-of-fit outweighs the slight reduction in generalization capacity from including additional predictors. It can be observed that the 2-parameter model presented the lowest LOOCV error across all formulations (MAE of 2.27). Considering this model as the baseline, the addition of non-pooled covariates improved predictions in the custard group (0.9–10.3% MAE improvement), but reduced them in the biscuit group (9.1–21.6% MAE deterioration), and overall, the generalization capacity was slightly reduced (4.8–5.7% MAE deterioration). On the other hand, goodness of fit improved between 7.2% and 31.3% across all formulations, especially for custards (up to 37.7% improvement).

**Figure 2:**
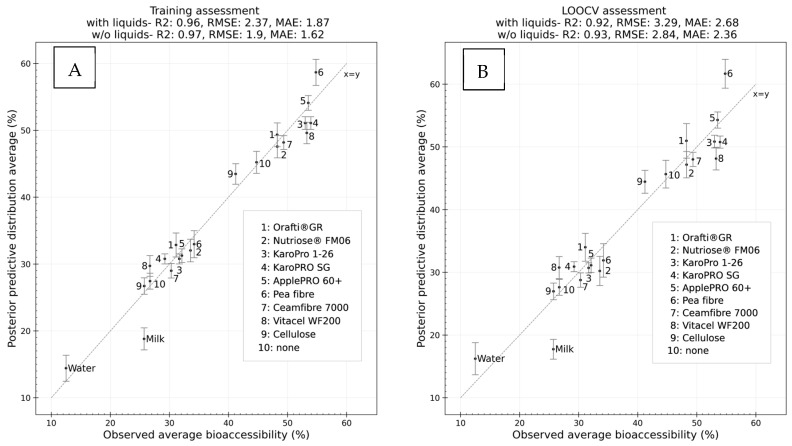
Training (**A**) and Leave-One-Out Cross-Validation (**B**) assessments of the goodness of fit and generalization capacity of the final model (8-parameter). Each dot corresponds to the average observed bioaccessibility of each formulation and is compared to the average of its posterior predictive distribution. The intervals shown for each point represent the 95% highest-density interval and are included to assess whether the credible interval correctly captures the observed averages (x=y line).

These results indicate that, for the purposes of training a model with the highest generalization capacity, a simple linear regression model using macronutrient content as the sole predictor is an acceptable option. If the goal is to attempt to ascertain the factors with the most influence on bioaccessibility in each matrix type, a Bayesian hierarchical model offers flexibility in terms of studying the most relevant features in each group whilst providing a measure of uncertainty of the importance of the predictors. In this case, the six-parameter model was considered to best balance prediction accuracy in both the training and validation samples. A more detailed representation of the goodness of fit and a simulation of the generalizability of the six-parameter model is visualized in Figure 2.

Regarding feature selection (Section 2.6.2), the autofeat and Boruta packages were deployed, which determined that the macronutrient content was sufficient to fit an accurate model in custards, although the weakly correlated particle size feature could assist in the case of linear modelling. In biscuits, it appeared that more features were necessary for accurate modelling, and autofeat concluded that the macronutrient content could even be substituted for a number of other features instead (a number large enough that the model would likely be overfitting). For the final feature selection using Laplacian priors, which was the configuration most similar to the final models presented in this work and therefore the most reliable in this context, the features that were relevant for both groups were those that had only been preselected in biscuits, although soluble fibre presented a significant correlation to custard bioaccessibility (ρ=0.38, *p*-value < 0.01), which supported its importance. On the other hand, Table 6 suggests that it was the out-of-sample prediction of custards which most benefitted from the inclusion of additional covariates.

Soluble fibre content was the non-pooled predictor to which the Laplacian prior model attributed the highest predictive power across both groups, and this is reflected in the six-parameter model’s posteriors (Table 7). Hemicellulose presented the strongest association with bioaccessibility (ρ=0.66, *p*-value < 0.01) of all the non-pooled predictor candidates, and this is also reflected by the model (6.2 ± 1.6 posterior). Every predictor presented a positive proportional association to observed bioaccessibilities and prediction outcomes. 

A heuristic interpretation from the fitted models is that increasing the concentration of nutrients in the formulation is the most significant factor leading to higher bioactive release from the matrix, and although there are interactions depending on the type of fibre being added to the formulation, the effect is small, and it is not yet possible to hypothesize a mechanical or molecular explanation for the underlying complexity with confidence. 

Figure 2 indicates that, although the model had a good fit to the training data ($r^{2}=0.97$) and formulations were acceptably inferred in the LOOCV experiment, with most formulations’ credible intervals capturing the observed average or presenting a relative error lower than 10%, some of the formulations’ observed bioaccessibilities were not properly captured. The clearest outliers were the biscuits formulated with fibre supplements composed purely of insoluble fibres or with a high content in such fibre (microcrystalline celulose and Vitacel WF200) and pea fibre (Table 2). None of these supplements present soluble fibre; hence, the prediction of their formulations is only affected by macronutrient and hemicellulose content. As Vitacel WF200 contains hemicellulose, whereas microcrystalline cellulose does not, it has correctly been deemed to have a higher bioaccessibility. The fact that hemicellulose presents a high correlation to biscuit bioaccessibility when insoluble fibre as a whole does not suggests the correlation might be spurious and that other factors might be influencing with a higher impact, but this is not observable when analysing bivariate correlations in this manner. There is plausibility for the existence of complex interactions occurring in the food matrix during digestion.

Nutriose custard was also poorly predicted in the LOOCV. Nutriose is a pure soluble fibre supplement like inulin; both display moderate bioaccessibility in biscuits, but nutriose presents a slightly higher bioaccessibility in custards. Inulin is a soluble fibre composed of fructans (polysaccharides of fructose), while nutriose is composed of resistant dextrin. The inclusion of dextrin covariate in the model could resolve this bias error; however, due to the highly sparse nature of this feature (only nutriose has dextrin), it can be considered as an identifier rather than an informative feature, and it was not included to favour the generalisation capacity. Although the features used in the model are features that can be applicable to many formulations, it is possible that, in the future, additional data can provide more insights into more appropriate features for the model, such as properties of the intermediate state of the food (i.e., biscuit dough texture) or the possibility of employing features that are currently sparse due to the limited number of formulations tested. In the case of this study, soluble fibre was a very informative feature, but it would not be useful for predicting formulations that do not include any fibres. Similarly, the need to prepare the formulations to include their texture properties in the model could be replaced by other features that do not require cooking the formulation beforehand. For example, the fibre supplement’s oil- and water-holding capacities and bulk densities presented significant correlations to curcuminoid bioaccessibility in both custards and biscuits, but as their value would have to be imputed for the case of formulations prepared without any supplements, their general applicability is impaired (after multiple imputation, they were not deemed as significant predictors according to the final selection). 

Modelling with a Bayesian hierarchical approach demonstrated great potential for predicting bioaccessibility for the study of formulations of not only custards and biscuits, but also of other matrix types, as it addresses the issue of imbalanced datasets through probabilistic modelling and the use of pooled covariates whilst still offering flexibility in including more factors explaining the variability in bioaccessibility within each food type. 

The choice of other relevant features for modelling bioaccessibility is less apparent, and yet, there is previous work supporting the claim that soluble fibre content is meaningful for modelling. In a study characterizing fusarotoxins enniatins’ (fat-soluble compounds) bioaccessibility in different bread formulations, 5% inulin supplementation reduced the bioaccessibilities of different enniatin compounds by half compared to what 10% of inulin affected, suggesting that the addition of different concentrations of fibre could provide proportional effects on bioaccessibility and could be a possible future opportunity for study, as well as other soluble fibres such as β-1,3 glucan and chitosan, which reduced enniatin bioaccessibility even further than inulin [45]. In a separate study, wheat bran with fibres (24% dietary fibre content compared to 5% on average of 13 other formulations) resulted in the lowest enniatin bioaccessibility when considering different breads, cereals, and cookies, although the fibre types were not specified [46]. This is in line with the general assumption that dietary fibres reduce the bioaccessibility of fat-soluble compounds such as curcuminoids due to altered diffusion of gastrointestinal fluids and even binding or trapping of bioactives, which would all result in a lesser degree of digestion and curcuminoid release [41,47,48,49]. The degree to which this could impact bioaccessibility in curcuminoids could be assessed more accurately with semi-dynamic [33] in vitro digestion systems to account for the different rates of gastrointestinal flow as a function of food matrix rheological/texture properties. Additionally, the experimental design should account for the possibility that released curcuminoids are absorbed by the body, thereby minimising oversaturation of curcuminoids in the chyme. Avoiding this oversaturation could increase the solubility and potentially the bioaccessibility, and so it should also be considered as an improvement over the current method for future analysis. Gastric emptying has been shown to significantly affect the accuracy of in vitro models [50]. 

The texture properties of the food matrix did not prove to be meaningful predictors for curcuminoid bioaccessibility compared to soluble fibre and hemicellulose (Table 6) Conducting experiments that account for gastrointestinal movements in a more realistic manner might shed more light on whether these inferences are accurate, as soluble fibre and hemicellulose are responsible for causing (at least partly) differences in food texture, especially in custards ($\rho >\left|0.70\right|$). 

The impact of macromolecules (proteins, carbohydrates, lipids and fibres) is highly correlated to curcumin bioaccessibility (Table 5), which suggests that their capacity to form or stabilize emulsions has a powerful effect on increasing the bioaccessibility of curcumin. It has been shown that curcumin can form complexes with proteins [51]. This complexation leads to an increase in curcumin’s aqueous solubility and bioaccessibility [52]. As previously mentioned, for lipophilic compounds like curcuminoids, the formation of emulsions with proteins, lipids, or both is a key factor improving bioaccessibility and bioavailability from food matrices [44]. Moreover, it was shown that increasing the concentration of sugar (sucrose) incorporated during the formation of milk protein/fat emulsions appeared to slightly decrease the droplet size that could impact the bioaccessibility. Indeed, it was shown that curcumin bioaccessibility increased with the decrease in droplet size for oil-in-water emulsions [53,54]. The concentration of sugar and fat is higher in biscuits compared to custards, potentially explaining a higher bioaccessibility for biscuits than for custards, even if the food matrix is more compact (Table S3). On another hand, soluble fibres such as pectins, when combined with egg proteins, have a stabilization effect on emulsions, increasing curcumin’s bioaccessibility [55].

Concerning insoluble fibres such as hemicellulose and cellulose, their impact on bioaccessibility could be explained by their capacity to form pickering emulsions due to their capacity to crystallize. These solid colloidal particles can adsorbed at the water/oil interface to stabilize it and exert a protecting effect on curcumin through the digestion process [56,57]. However, we did not monitor the formation of these type of emulsions in this study. The impact of insoluble dietary fibres on curcumin bioaccessibility could be also be more simply explained by direct interactions leading to an impact on bioaccessibility [58], although curcumin’s binding capacity to insoluble fibres appears not to be a significant predictor of its bioaccessibility, either for custards or biscuits (Figure S5).

Previous modelling attempts of oral bioaccessibility/bioavailability have not focused on the systematic optimisation of an individual compound as a function of formulation. To clarify, the study of bioaccessibility/bioavailability can also be conducted based on the observation of its practical applications: how biorelevant a compound’s antioxidant capacity remains after the digestion process [59] or how much response to treatment a compound elicits when ingested, which is the main focus of most studies in the field, as they usually pertain to drug development. A large majority of previous works have generally targeted the modelling of several compounds at once using data from existing databases, and have shown varying degrees of success. The modelling of bioavailability is generally addressed as a classification problem, which enables higher performances to be achieved at the cost of a loss of information. Degrees of accuracy typically range from 60% to 97%, with 70–80% on average, for example, for goals as unassuming as estimating whether a drug has a positive, negative, or inexistent food effect [29,60,61,62,63]. The descriptors are typically molecular properties of each drug derived from concepts such as its membrane permeation, metabolic stability, solubility, acidity, and lipophilicity. In the case of focusing on a single compound as a function of formulation, identifying whether the formulations have a significant impact on these properties which would consequently alter their bioaccessibility/bioavailability is a potential approach for systematically modelling compounds individually as well. In addition, the notion that the evidence from one compound can aid in inferring the bioaccessibility/bioavailability of another compound is promising. Another group of modelling studies regarding oral bioaccessibility/bioavailability is directed towards risk assessment to estimate the body burden of different contaminant compounds (such as lead or mercury) on dietary exposure by approximation with Bayesian methods [64,65]. 

According to our review of the existing literature to date, another study that resembles the work presented herein was published recently by Hernández-Prieto et al. in 2023. Urine samples from 140 volunteers during a 2-month period were analysed for polyphenolic metabolites after consumption of a maqui-citrus beverage that had been enriched with a sweetener (sucrose, sucralose, or stevia). Their results indicated that the sex of the volunteer, the sweetener, and their interaction significantly affected the bioavailability of the phenolic compounds. Therefore, this study can be considered as a non-exhaustive evaluation of the impact of food formulation on bioavailability (as a function of the sweetener), but it also evaluated whether the subject played a key role in the availability of the compound. Their findings indicated that the subject is an important factor for the modelling of absorption and should be considered in the experimental design whenever possible, which would increase the complexity of the analysis. Machine learning techniques were used to cluster the samples into six groups according to metabolic profiles; however, a predictive model was not proposed [66]. It appears that our case study is the first work attempting to build a predictive model of a compound’s bioaccessibility/bioavailability as a function of the food formulation exclusively.

The results showcase the potential usefulness of modelling bioaccessibility screening experiments of different food formulations. The known properties of the food matrix were used to infer the impact on bioaccessibility of curcuminoids. The test results indicated that, although the validation strategy used to test the aptitude of the model’s predictions was optimistic, the majority of the formulations, with some exceptions, were estimated acceptably, with a relative error lower than 10%. The fact that increased macronutrient concentrations are very strongly correlated to an increase in curcuminoid bioaccessibility could be used to inject prior knowledge into future models, and this is the first step in creating a prior belief system in this domain that could be applicable to other compounds as well to build cost-effective decision support systems for the optimization of functional foods. It should be noted, however, that modelling individual bioaccessibility in relation to the individual’s age, gender, and microbiome profile is beyond the predictive capability of the presented model. For instance, it has been shown in the literature that the gut microbiota impacts the absorption of minerals such as calcium [67], magnesium [68], and iron [69], and, inevitably, bioactives in general. However, predicting the impact of different food matrices on bioavailability will provide assistance as a decision-support system for designing formulations for optimum intake of bioactives.

Modelling with a Bayesian hierarchical approach has the potential to study formulations other than custards and biscuits, as it addresses the issue of unbalanced datasets through probabilistic modelling and the use of pooled covariates whilst still offering flexibility in including more factors explaining the variability in bioaccessibility within each food type. Finally, the insights from this model could be extrapolated to other compounds. Finally, the insights from this model could be extrapolated to other compounds by modelling not only as a function of food formulation but also of compound properties, in a multi-compound approach such as those previously conducted. The utility of a model such as the previously formulated one could be immense for aiding in the design of a wide range of optimal functional foods. The insights from these models could later be corroborated with bioavailability studies for the most promising formulations. 

## 4. Conclusions

This study investigated whether the bioaccessibility of a bioactive compound can be statistically modelled based on the food matrix characteristics. For this purpose, curcuminoids were the subject compound and were added to fibre-enriched custards and biscuits. The findings revealed significant differences in bioaccessibility among the formulations, with the matrix type and the interaction with fibre supplements playing key roles. The Bayesian hierarchical model developed in this study demonstrated good fit to the training data and provided valuable insights into the factors influencing bioaccessibility. The results underscored the importance of considering the complex interactions within food matrices and the specific properties of each formulation in understanding bioaccessibility. The study highlighted the potential of systematic modelling approaches for optimizing the design of functional foods. Future research can further explore additional features and refine the model to enhance its generalization capacity. Overall, this study contributes to the understanding of curcuminoid bioaccessibility and offers a framework for studying other formulations beyond custards and biscuits, and eventually other bioactive chemicals. Multi-compound models aiming to be cost-effective, such as those previously based exclusively on in silico molecular properties, could benefit from this approach.

## Figures and Tables

**Table 1:** Ingredients of standard and curcumin-enriched custards and biscuits.

Ingredients	Custard	Biscuit	Fibre-Fortified Custard	Fibre-Fortified Biscuit
Semi-skimmed milk (%)	68.3	4.1	64.1	3.8
Egg yolk (%)	13.2	39.9	12.6	37.7
Sugar (%)	10.6	32	10.1	30.2
Corn starch (%)	7.9	-	7.5	-
T45 Wheat flour (%)	-	24	-	22.6
Fibre (%)	-	-	5.7	5.7

**Table 2:** Supplemented fibre sources and their nutritional composition as presented in the products’ specifications provided by suppliers. Empty cells represent non-significant values.

	Insoluble Fibre (%)	Soluble Fibre (%)	Protein (%)	Carbohydrates (%)	Total Fat (%)	Water (%)	Ash (%)	Cellulose (%)	Hemicellulose (%)	Pectin (%)	Fructans (%)	Lignin (%)	Dextrin (%)	Origin
Orafti^®^ GR	0	>90	-	<10	-	3	<0.2	-	-	-	>90	-	-	Chicory
Nutriose^®^ FM06	-	82–88	<0.3	<0.5	0.1	<5	<0.5	-	-	-	-	-	82–88	Maize
FST 00007 KaroPRO 1-26	33	27	5	21	1	6	4	41	7	5	-	7	-	Carrot
FST 00018 KaroPRO SG														
Pea fibre 50 M	>50	-	<10	35	-	<10	3	20	30	-	-	-	-	Yellow pea
FST 00224 ApplePRO 60+	52	8	5.8	27.6	<3.2	<8	1.8	27	16	6	-	10	-	Apple
Ceamfibre 7000	85	1.5	2.9	-	0.8	7.6	-	41	16	1.5	-	6	-	Citrus peel
Vitacel WF200	97	-	0.4	-	0.2	<8	<3	24	73	-	-	-	-	Wheat
Microcrystalline cellulose	100	-	-	-	-	-	-	100	-	-	-	-	-	Wood pulp

**Table 3:** Measured physicochemical properties of the fibres employed in this study.

Fibre	Liquid-Holding Capacities (mL/Gram)		Densities (g/cm^3^)		Colour Measurements					Curcuminoid-Binding Capacity (mmol)	*D*_4.3_–De Brouckere Mean (µm)	Specific Surface Area (m^2^/g)
	Oil	Water	Bulk	Tapped	L	a	b	c	h			
Inulin (Orafti^®^GR)	2.2 ± 0.1	- *	0.60	0.65	93 ± 1	−11 ± 1	19 ± 1	22 ± 1	119 ± 2	- *	121 ± 63	0.15 ± 0.02
Nutriose FM06	2.8 ± 0.1	- *	0.38	0.45	89 ± 2	6 ± 1	18	19 ± 1	73 ± 2	- *	147 ± 15	0.15 ± 0.02
FST 00007 KaroPRO 1-26	4.3	8.7 ± 1.7	0.17	0.23 ± 0.10	85 ± 1	9	24 ± 1	25 ± 1	70	100	255 ± 3	0.04 ± 0.01
FST 00018 KaroPRO SG	3.0 ± 0.2	6.1 ± 0.1	0.33	0.54 ± 0.05	65 ± 1	18	33	38	61	100	37	0.28
Pea fibre 50 M	2.5 ± 0.1	4.2 ± 0.5	0.47	0.65 ± 0.03	72 ± 3	17 ± 1	28 ± 2	33 ± 2	59 ± 1	40	89 ± 2	0.17
FST 00224 ApplePRO 60+	2.3 ± 0.1	5.1 ± 0.6	0.41	0.69 ± 0.05	47 ± 2	23 ± 1	37	43	58 ± 1	320 ± 10 **	65 ± 4	0.41 ± 0.01
Ceamfibre 7000	4.2 ± 0.1	4.0 ± 0.6	0.21	0.30 ± 0.09	60	18	37	41	64	460 ± 70 **	262 ± 4	0.03
Vitacel WF200	6.3 ± 0.1	8.2 ± 0.5	0.12	0.23 ± 0.10	89 ± 2	5 ± 1	16 ± 1	17 ± 1	72 ± 5	220 ± 30 **	105 ± 1	0.13
Microcrystalline cellulose	3.0	2.2 ± 0.1	0.37	0.47	68 ± 1	17	25	30 ± 1	57	260 ± 10 **	91 ± 1	0.14

Cells without standard deviations correspond to a standard deviation of 0. *: Empty cells correspond to non-measurable values: curcuminoid-binding capacity and water-holding capacity of soluble fibres cannot be measured as the fibres dissolve in aqueous solutions. **: dispersions are indicative, not standard deviations.

**Table 4:** Measured textural properties of the fibre-fortified foods employed in this study.

Fibre	Biscuit Hardness (g)	Custard Viscosity (mPa·s)	Custard Firmness (g)	Custard Stickiness (g)
Inulin (Orafti^®^GR)	1802 ± 711	5383 ± 442	68 ± 22	−33 ± 10
Nutriose FM06	1612 ± 633	4242 ± 679	43 ± 15	−19 ± 6
FST 00007 KaroPRO 1-26	2075 ± 1573	sat. *	318 ± 16	−166 ± 23
FST 00018 KaroPRO SG	2157 ± 422	15,381 ± 1467	213 ± 21	−114 ± 14
FST 00224 ApplePRO 60+	1229 ± 229	sat. *	297 ± 14	−135 ± 17
Pea fibre 50 M	1419 ± 622	sat. *	506 ± 23	−303 ± 24
Ceamfibre 7000	3039 ± 1437	sat. *	354 ± 33	−152 ± 2
Vitacel WF200	3028 ± 675	17,963 ± 1231	250 ± 17	−135 ± 12
Microcrystalline cellulose	2362 ± 660	13,659 ± 1712	166 ± 12	−90 ± 13
none	1709 ± 285	7453 ± 1608	91 ± 55	−60 ± 43

Cells without standard deviations correspond to standard deviation of 0. *: 25,000 mPa·s was the saturation limit of custard viscosity measurements.

**Table 5:** Candidate explanatory variables’ correlation to bioaccessibility and algorithms supporting their inclusion in the model.

		All Formulations	Custards	Biscuits
Fibre properties	Oil-holding capacity	n.a.	–0.39 **	–0.10 (1)
	Water-holding capacity		–0.14	–0.47 ** (2)
	Bulk density		–0.39 **	–0.12
	Curcumin-binding capacity		–0.19	–0.37 (2)
	Particle size		–0.14 (1)	–0.31 *
Matrix properties	Biscuit hardness		n.a.	–0.17
	Custard viscosity		–0.05	n.a.
	Custard firmness		–0.06	
	Custard stickiness		–0.07	
	Cellulose		–0.41 ** (1)	–0.15
	Hemicellulose		–0.12	–0.66 ** (1, 2, 3)
	Pectin		–0.10	–0.45 ** (1)
	Insoluble fibre		–0.31	–0.14 (1)
	Soluble fibre		–0.38 ** (3)	–0.0 (1, 3)
	Protein	–0.89 **	–0.36 **	–0.72 **
	Carbohydrate	–0.89 **	–0.46 **	–0.65 **
	Fat	–0.84 **	–0.12	–0.52 **
	Water	–0.62 **	–0.30 *	–0.72 **
	Ash	–0.72 **	–0.23	–0.70 **
Feature-engineered matrix properties	Macronutrient	–0.89 **	–0.47 ** (1, 2, 3)	–0.66 ** (2, 3)

Spearman rho (ρ) coefficients were used as a correlation metric. These were grouped by matrix type and computed for all formulations simultaneously. Properties selected for modelling in one matrix type are indicated by numbers in parentheses next to their coefficients (1: selected by autofeat, 2: selected by Boruta, 3: final selection by Laplacian priors). Macronutrient content was the only nutritional composition feature considered during selection, as its component features (carbohydrate, fat, protein) presented high collinearity. n.a.: non-applicable. *: *p*-value between 0.01 and 0.05 **: *p*-value under 0.01.

**Table 6:** Performance metrics of several Bayesian hierarchical models of curcuminoid bioaccessibility built with different feature combinations.

Model Complexity (n Parameters)	Additional Predictors	Training Performance									LOOCV Performance								
		Custard Formulations			Biscuit Formulations			All Formulations			Custard Formulations			Biscuit Formulations			All Formulations		
		$r^{2}$	MAE	RMSE	$r^{2}$	MAE	RMSE	$r^{2}$	MAE	RMSE	$r^{2}$	MAE	RMSE	$r^{2}$	MAE	RMSE	$r^{2}$	MAE	RMSE
2	-	0.31	2.04	2.33	0.53	2.12	2.92	0.94	2.08	2.64	0.17	2.23	2.56	0.46	2.31	3.13	0.93	2.27	2.86
4	+soluble fibre	0.57	1.74	1.85	0.53	2.13	2.91	0.95	1.93	2.44	0.27	2.21	2.40	0.41	2.52	3.28	0.93	2.36	2.88
6	+hemicellulose	0.70	1.37	1.53	0.73	1.87	2.23	0.97	1.62	1.91	0.34	2.00	2.29	0.38	2.77	3.36	0.93	2.38	2.88
8	+food texture	0.73	1.27	1.47	0.80	1.59	1.6	0.97	1.43	1.70	0.29	2.00	2.37	0.37	2.81	3.39	0.92	2.40	2.92

**Table 7:** Posteriors of the 6-parameter model.

Parameter		Mean ± Std	HDI Lower	HDI Upper
Intercept		14.3 ± 1.0	12.5	16.1
Macronutrient content		37.8 ± 1.7	34.9	41.3
Soluble fibre	Custards	5.1 ± 1.2	3.0	7.4
	Biscuits	3.2 ± 1.3	0.9	5.5
Hemicellulose	Custards	3.1 ± 1.2	0.8	5.4
	Biscuits	6.2 ± 1.6	3.0	9.2

HDI: highest-density interval: 3% to 97%.

## Data Availability

Data generated as part of this work and the associated analysis scripts are publicly available at: https://github.com/FadyMohareb/cbioaccessibility.

## Supplementary Materials

The following supporting information can be downloaded at: https://www.mdpi.com/article/10.3390/foods13142234/s1, Figure S1: HPLC separation with UV 430 nm detection, full separation (1) and zoom (2) for (A) curcuma standard 4 mg/L, (B) Biscuit without curcumin and (C) Biscuit with curcumin after digestion; Figure S2: UV430 nm spectrum for CUR (a), UV 430 nm chromatogram (B) and extracted ion chromatogram at 307.0975, 337.1091, 367.1187 uma for BDMC (C), DMC (D), and CUR (E), respectively. LC-ESI(-)-HRMS for BDMC (F), DMC (G), and CUR (H); Figure S3: principal component analysis (PCA) of all candidate predictors of curcuminoid bioaccessibility, excluding fibre supplement colour properties. Predictors were standardised prior to singular value decomposition; Figure S4: principal component analysis (PCA) of the predictor subset employed in the 6-parameter model. Predictors were standardised prior to singular value decomposition; Figure S5: Measured curcuminoid binding capacities. Measurements belong to experiments performed on separate occasions. The curcuminoid binding capacity attributed to each fibre was the highest added curcuminoid concentration (average) under the 2.5 mM threshold (intended as a relative measure); Table S1: Candidate predictors of curcuminoid bioaccessibility; Table S2: summary of 3 main curcuminoids from turmeric curcumin; Table S3: Composition in proteins, fat, and carbohydrates of the fibre-enriched custards and biscuits according to product labelling and literature imputation.
