# Supplementary material for: Probabilistic Modelling of the Food Matrix Effects on Curcuminoid’s In Vitro Oral Bioaccessibility
Source: Foods. 2024 Jul 16;13(14):2234. doi: 10.3390/foods13142234 (PMC11276217; doi:10.3390/foods13142234)
Supplement: Supplementary file 1 [file foods-13-02234-s001.zip › foods-3078149-supplementary.pdf]

Article

Probabilistic modelling of the food matrix effects on curcuminoid’s oral bioaccessibility

Kevin de Castro Cogle [1,2], Mirian T.K. Kubo [1], Franck Merlier [1], Alexandra Josse [1], Maria Anastasiadi [2], Fady R. Mohareb [2,*], Claire Rossi[1,*]

[1] Université de technologie de Compiègne; CNRS; UPJV; GEC; Compiègne; France.
[2] Bioinformatics Group; Centre for Soil; Agrifood and Biosciences (SABS); Cranfield University; College Rd; Cranfield; Bedford MK43 0AL; United Kingdom
* Correspondence: FRM: f.mohareb@cranfield.ac.uk; CR: claire.rossi@utc.fr.

Supplementary Figures

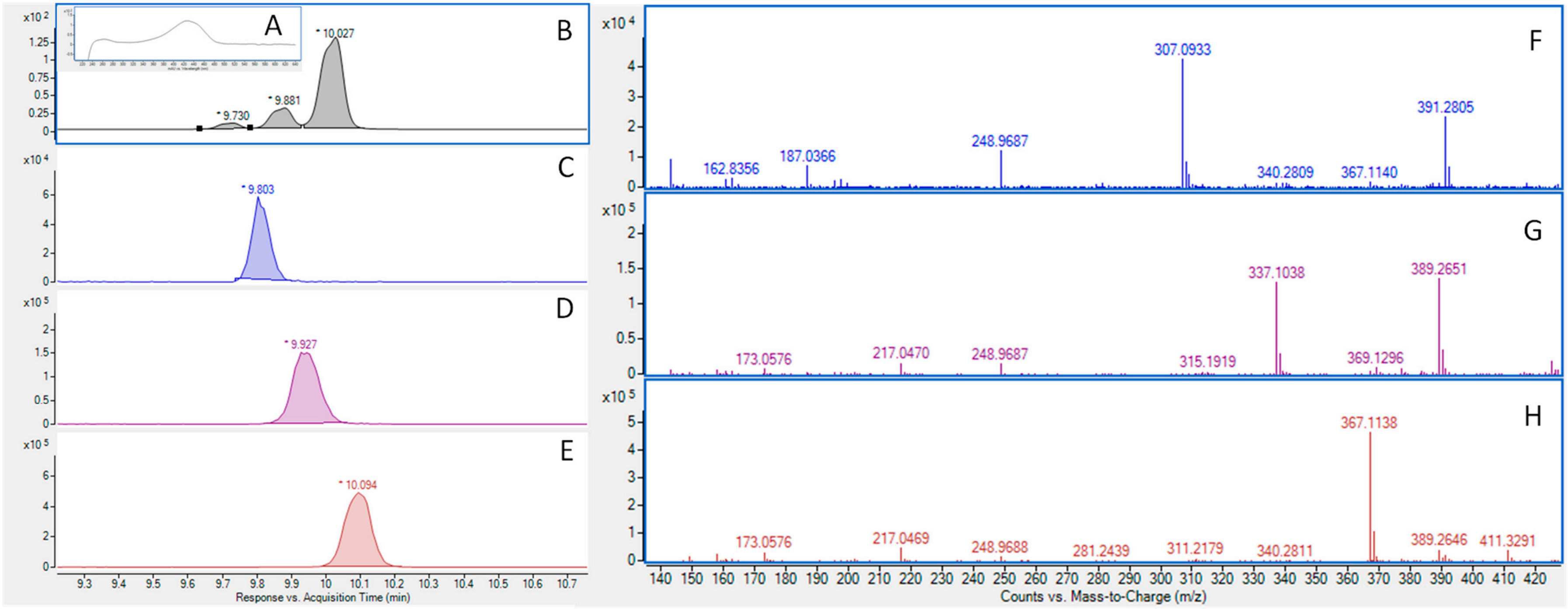

Figure S1: UV430 nm spectrum for CUR (a), UV 430 nm chromatogram (B) and extracted ion Chromatogram at 307.0975, 337.1091, 367.1187 uma for respectively BDMC (C), DMC (D) and CUR (E). LC-ESI(-)-HRMS for BDMC (F), DMC (G) and CUR (H).

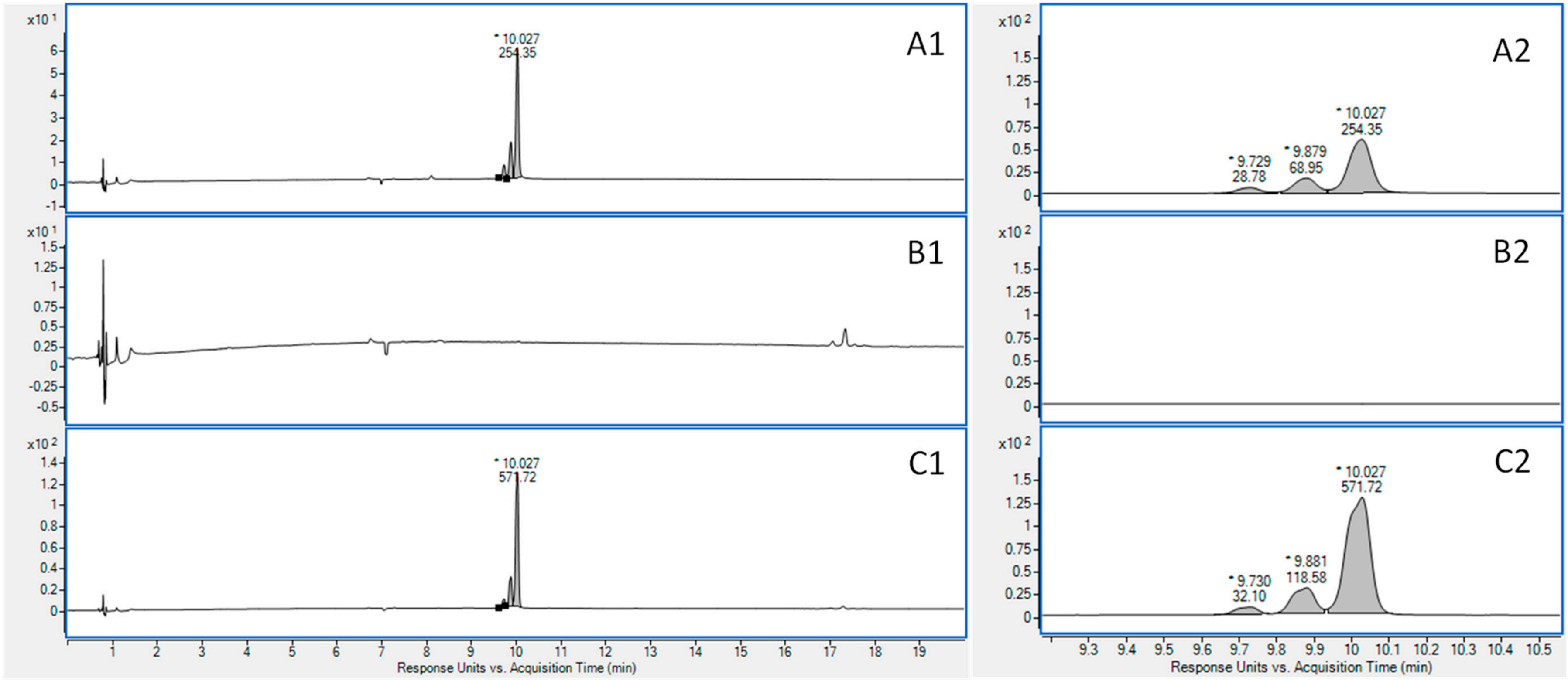

Figure S2 : HPLC separation with UV 430 nm detection , full seperation (1) and zoom (2) for (A) curcuma standard 4 mg/L, (B) Biscuit without curcumin and (C) Biscuit with curcumin after digestion

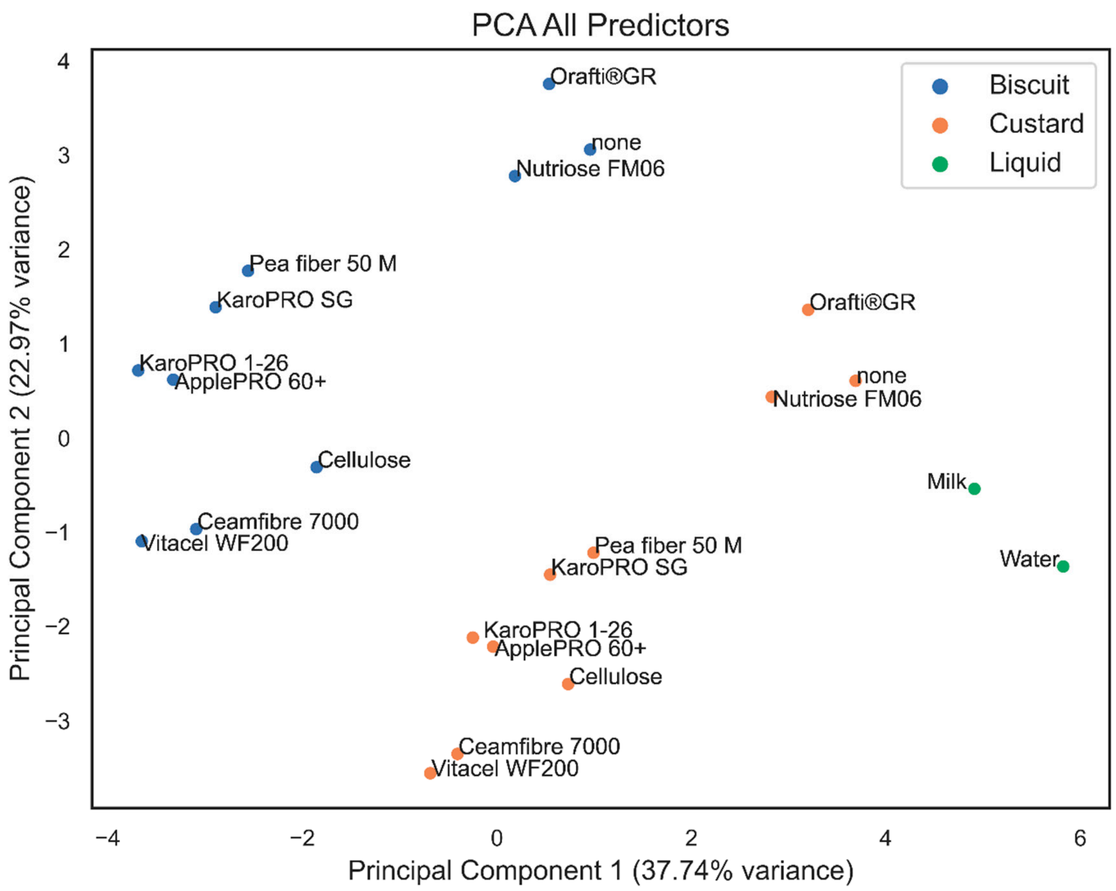

Figure S3. Principal Component Analysis (PCA) of all candidate predictors of curcuminoid bioaccessibility, excluding fibre supplement colour properties. Predictors were standardised prior to Singular Value Decomposition.

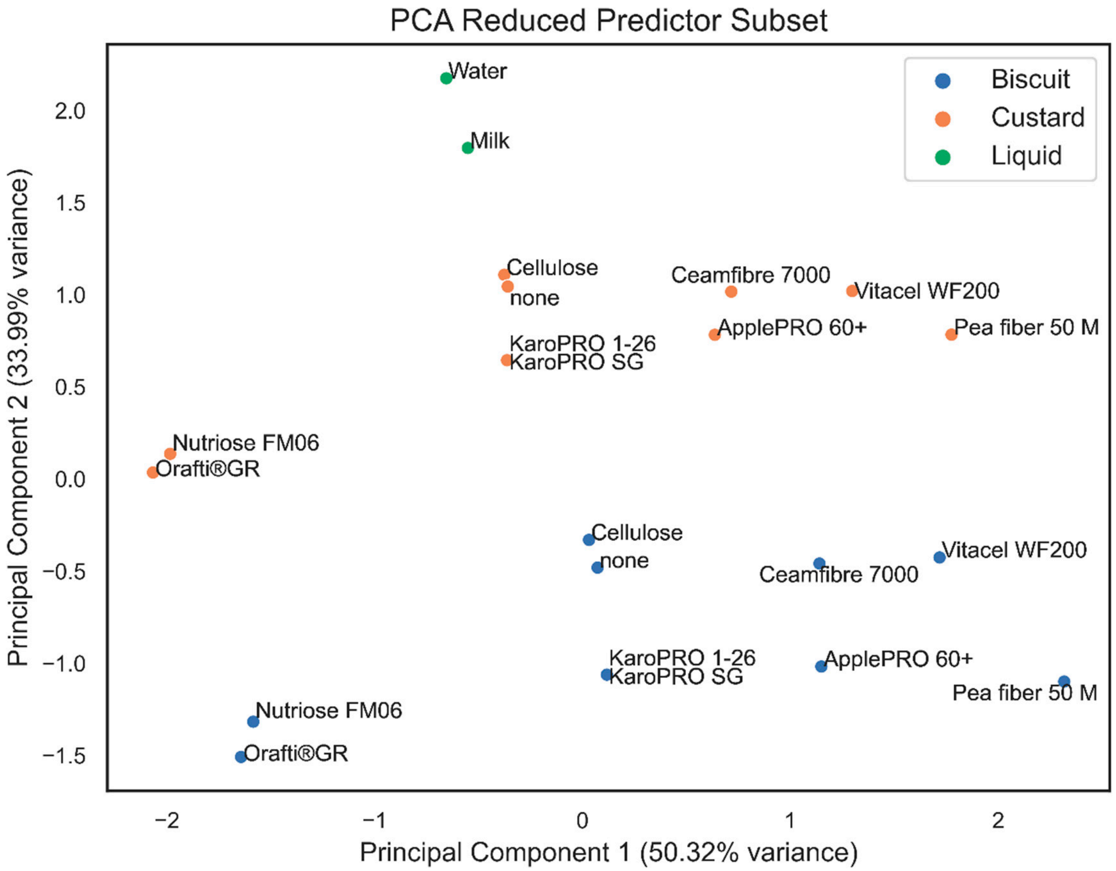

Figure S4. Principal Component Analysis (PCA) of the predictor subset employed in the 6-parameter model. Predictors were standardised prior to Singular Value Decomposition

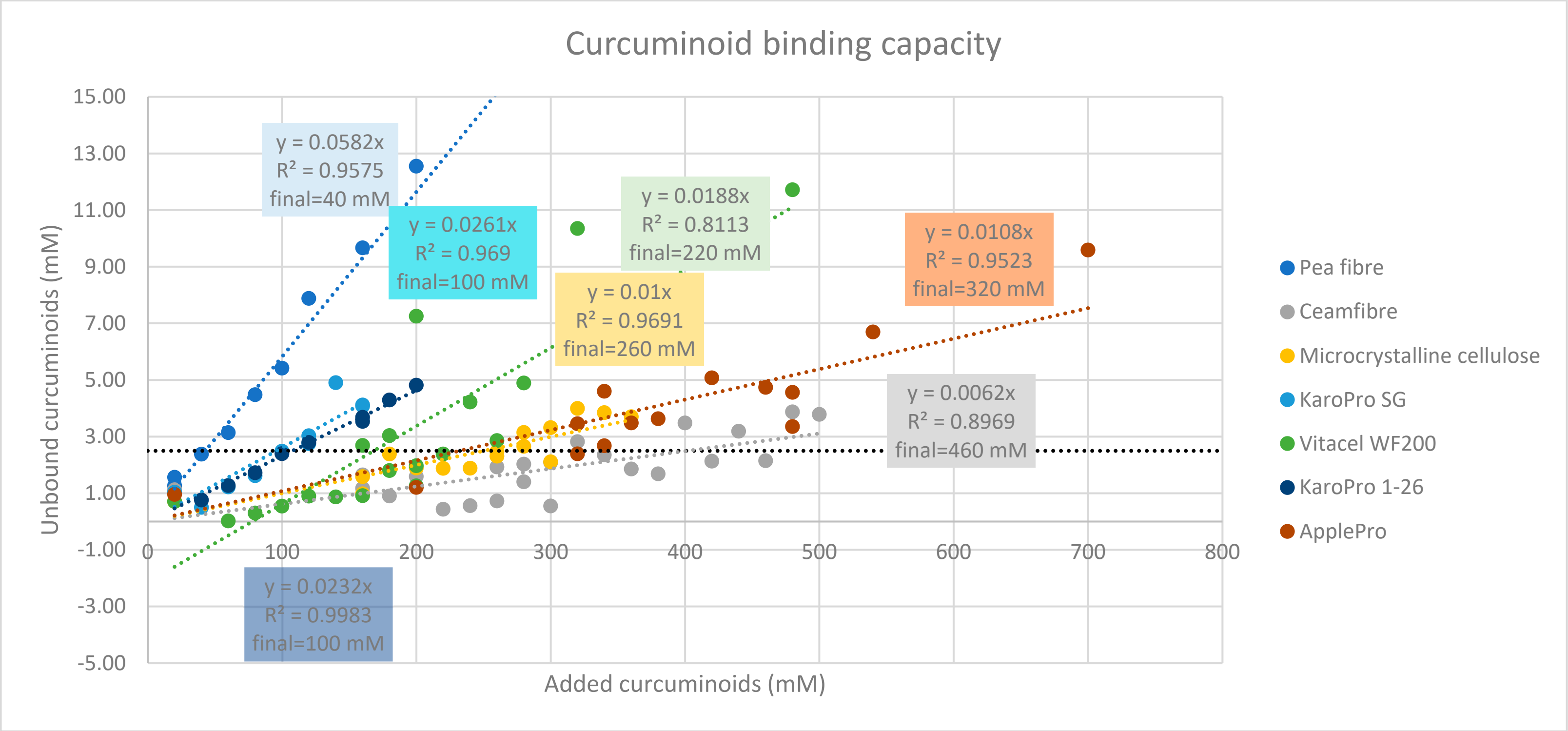

Figure S5: Measured curcuminoid binding capacities. Measurements belong to experiments performed on separate occasions. The curcuminoid binding capacity attributed to each fibre was the highest added curcuminoids concentration (average) under the 2.5 mM threshold (intended as a relative measure).

Supplementary Tables

Table S1: Candidate predictors of curcuminoid bioaccessibility.

**Formulation descriptors**	**Nutritional composition content**	Soluble fibre	Insoluble fibre	Protein	Carbohydrates	Fat	Water	Ash
	**Fibre type**	Cellulose	Hemicellulose	Dextrin	Pectin	Lignin	Fructans	
**Supplement descriptors**	**Physicochemical properties**	Water holding capacity	Oil holding capacity	Bulk density	Tapped density	Particle size diameter	Particle surface area	Curcuminoid binding capacity
	**Colour**	L	a	b	C	h		
**Matrix type descriptors**	**Custards**	Viscosity	Firmness	Stickiness				
	**Biscuits**	Breaking force						

Table S2 : summary of 3 main curcurmoids from turmeric curcumin

Molecule	Raw Formula	Mass Weight (g/mol)	[M-H]- (uma)	Retention time (min)
Bis-Demethoxy curcumin (BDMC)	$C_{19}H_{16}O_4$	308.10	307.0975	9.80
Demethoxy curcumin (DMC)	$C_{20}H_{18}O_5$	338.12	337.1081	9.93
Curcumin (CUR)	$C_{21}H_{20}O_6$	368.13	367.1187	10.09

Table S3. Composition in proteins, fat and carbohydrates of the fibre-enriched custards and biscuits according to product labelling and literature imputation.

	Custards					Biscuits				
	**Protein (%)**	**Carbohydrates**		**Fat**		**Protein (%)**	**Carbohydrates**		**Fat**	
		**Total (%)**	**Sugars (%)**	**Total (%)**	**Saturated (%)**		**Total (%)**	**Sugars (%)**	**Total (%)**	**Saturated (%)**
Orafti® GR	3.60	20.82	13.77	3.99	0.65	6.76	51.89	31.36	9.35	0.10
Nutriose® FM06	3.64	19.64	13.23	4.00	0.65	6.87	48.30	30.82	9.39	0.10
FST 00007 KaroPRO 1-26<br>FST 00018 KaroPRO SG	4.23	22.20	13.20	4.11	0.67	8.65	56.04	30.79	9.73	0.12
Pea fibre 50 M	4.85	23.95	13.20	3.99	0.65	10.53	61.32	30.79	9.35	0.10
FST 00224 ApplePRO 60+	4.32	23.02	13.20	4.39	0.69	8.95	58.53	30.79	10.56	0.13
Ceamfibre 7000	3.96	19.58	13.20	4.09	0.65	7.85	48.11	30.79	9.65	0.10

Vitacel WF200	3.65	19.59	13.20	4.01	0.65	6.91	48.15	30.79	9.43	0.10
Microcrystalline cellulose	3.60	19.58	13.20	3.99	0.65	6.76	48.11	30.79	9.35	0.10
Non-supplemented	3.81	20.73	13.99	4.22	0.69	7.16	50.98	32.62	9.91	0.10
